# Distributed Environment Control Using Wireless Sensor/Actuator Networks for Lighting Applications

**DOI:** 10.3390/s91108593

**Published:** 2009-10-28

**Authors:** Masayuki Nakamura, Atsushi Sakurai, Jiro Nakamura

**Affiliations:** NTT Energy and Environment Systems Laboratories, Nippon Telegraph and Telephone Corporation, Morinosato Wakamiya, Atsugi-shi, Kanagawa Pref., Japan; E-Mails: a2c@aecl.ntt.co.jp (A.S.); jnaka@aecl.ntt.co.jp (J.N.)

**Keywords:** sensor/actuator network, distributed control, decentralized system

## Abstract

We propose a decentralized algorithm to calculate the control signals for lights in wireless sensor/actuator networks. This algorithm uses an appropriate step size in the iterative process used for quickly computing the control signals. We demonstrate the accuracy and efficiency of this approach compared with the penalty method by using Mote-based mesh sensor networks. The estimation error of the new approach is one-eighth as large as that of the penalty method with one-fifth of its computation time. In addition, we describe our sensor/actuator node for distributed lighting control based on the decentralized algorithm and demonstrate its practical efficacy.

## 1. Introduction

Wireless sensor/actuator networks (WSANs) have been investigated in addition to wireless sensor networks (WSNs) since WSANs have more attractive and useful applications than WSNs alone. In WSNs, the main objective is to gather raw sensor data or estimate the condition of the environment. From such a sensing standpoint, Zhao *et al.* proposed collaborative signal and information processing (CSIP) to target tracking problems [1]. They showed that collaborative and decentralized sensor networks are scalable and efficient as regards sensing and communication. Rabbat *et al.* investigated distributed algorithms for sensor network data processing [2]. They formulated estimation problems as optimization problems in distributed WSNs. WSANs have the potential to expand WSN applications, enabling the nodes to perform both sensing and actuation [3-5]. A promising WSAN application is the control of spatially distributed actuators, such as dimmable lighting ballasts, switches and air conditioning systems. WSANs can save energy because they can accurately monitor environmental conditions and thus control the actuators precisely. In this context, Taylor *et al.* developed WSANs for energy management in heating, ventilation, and air-conditioning (HVAC) systems [6]. Zhang *et al.* proposed a field estimation technique that uses WSANs to control HVAC systems in buildings [7]. Li *et al.* developed WSANs for lighting control in the home environment [8]. One challenge is to develop algorithms that save energy without sacrificing user comfort. Sandhu *et al.* proposed WSANs for lighting control using a multi-agent system [9]. Singhvi *et al.* developed a centralized lighting system to increase user comfort and reduce energy costs by using Motes [10]. Lin *et al.* proposed a decentralized algorithm for WSANs for optimal lighting control [11]. We proposed WSANs that can provide optimal actuator control with respect to energy saving and control signal quality as well as sensing [12]. The sensor/actuator nodes perform sensing and actuation autonomously. However, the decentralized algorithm is based on the penalty function method, and it takes a long time to compute optimal control signals.

In this paper, we introduce an improved collaborative sensing and actuation algorithm in an optimization framework for controlling lights in workplaces. In our algorithm, an objective function is defined that balances energy saving against control signal quality. We describe a decentralized algorithm that is more scalable than the centralized one, and that can autonomously calculate control signals without a central server. This algorithm uses an appropriate step size in the iterative process for calculating control signals. We demonstrate its accuracy and efficiency compared with the previously proposed method by simulations. We also carry out WSAN experiments using Motes to examine the feasibility of the algorithm. We show that the estimation error of the proposed method is one-eighth as large as that of the previous method with one-fifth of its computation time. In addition, we describe a testbed that consists of Motes and infrared (IR) remote controls for distributed lighting control based on the decentralized algorithm.

## 2. Distributed WSAN Model

In dense distributed WSANs, collaborative processing is essential for intelligent sensing and for controlling environments such as shared workplaces. To conserve energy, local sensing usually determines the local actuation of, for example, a light by using an occupancy sensor. When sensor/actuator nodes are networked, the quality of the control signals within the WSANs is improved, resulting in occupant satisfaction. In this paper, we use “control signal” as the signal applied to actuators. We assume that a control signal, for example a current, corresponds to the controlled environmental condition, such as the brightness of lights. We focus on the energy saving of the lights. In addition, the spatial smoothness of brightness is likely to be preferred by users. We propose a method that balances energy saving and the spatial smoothness of the control signals to improve control signal quality. Our method is formulated as an optimization problem. Let *J* be an objective function defined as:

(1) $$J=\frac{1}{2}\sum_{i=1}^{n}(\frac{\alpha }{2}\sum_{j\in N_{i}}{\left(f_{i}-f_{j}\right)}^{2}+(1-\alpha )f_{i}^{2}),$$

where *n* is the number of sensor nodes, *f_i_* is the control signal of sensor node *i, f_j_* is the control signal of sensor node *j* within the communication range *N_i_* of sensor node *i*, and *α* (0 < *α* < 1) is a tradeoff parameter balancing $\frac{1}{2}\sum_{i=1}^{n}(\frac{1}{2}\sum_{j\in N_{i}}{\left(f_{i}-f_{j}\right)}^{2})$ in Equation (1) (the spatial roughness of the control signals) against $\frac{1}{2}\sum_{i=1}^{n}f_{i}^{2}$ (the consumed energy). For this discussion, let us consider that the sensors are passive infrared (PIR) occupancy sensors that detect the presence of people, and that the controlled actuators are lights. Occupancy sensor response *s_i_* is binary such that:

(2) $$s_{i}=\{\begin{array}{l} 1\;(\text{detect})\\ 0\;(\text{not detect})\;. \end{array}$$

The lights are turned on in areas where people are present. To simplify the problem, we assume that the control signals are set at 1 where people are present, that is:

(3) $$f_{i}=1\;\text{if}\;s_{i}=1$$

Minimizing *J* with respect to *f_i_* means that lights must be turned on in areas where people are present and controlled to balance the spatial smoothness of the control signals and energy saving otherwise. To obtain *f_i_* that minimizes *J*, the partial derivative of *J* with respect to *f_i_* is calculated as:

(4) $$\partial J/\partial f_{i}=\alpha \sum_{j\in N_{i}}(f_{i}-f_{j})+(1-\alpha )f_{i}=0\;\text{if}\;s_{i}\;=0.$$

Equation (4) is a simultaneous equation which represents a centralized algorithm. It can be solved by a server collecting sensor responses from all sensor nodes. After collecting the sensor responses { *S*_1_, ‥, *S_i_*_−1_, *S*_1_, *S_i_*_+1_, ‥, *S_n_* }, the server computes control signals { *f*_1_, ‥, *f_i_*_−1_, *f*_1_, *f_i_*_+1_, ‥, *f_n_* } by solving Equation (4) and sends the resulting control signals back to the sensor nodes. The sensor nodes control their external actuators through their digital output ports depending on the control signals.

It is also possible to use a decentralized algorithm, which can compute each control signal without a server. Rabbat *et al.* showed that distributed optimization algorithms are more efficient than centralized ones in terms of energy and communications [2]. The gradient method can be used to determine the value of *f_i_* that minimizes *J* [13]. Using the gradient method, *f_i_* is incrementally updated by Δ*f_i_* as:

(5) $$\begin{array}{ll} \Delta f_{i} & =-\varepsilon \partial J/\partial f_{i}\\ & =-\varepsilon (\alpha \sum_{j\in N_{i}}\left(f_{i}-f_{j})+(1-\alpha )f_{i}\right)\;\text{if}\;s_{i}\;=0, \end{array}$$

where *ε* is a positive step size.

Formerly *J* was redefined to include the condition of Equation (3) using the penalty function method [12]. This approach was very useful for formulating the objective function without classifying the values of *s_i_*. However, the convergence of *f_i_* was very slow. In this paper, *f_i_* is computed separately by Equations (3) and (5) depending on the values of *s_i_*.

**Figure 1.**
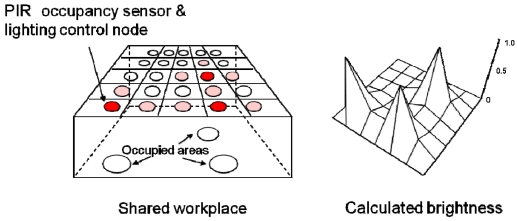
Decentralized sensor/actuator network system and calculated brightness of each node.

Figure 1 shows a decentralized sensor/actuator network system in a shared workplace and an example of calculated brightness for each node. Each sensor node is arranged at regular intervals and communicates with neighboring nodes to compute its control signal locally. In this model, there is no central server collecting sensor data and computing control signals. It is necessary to repeat the updates described by Equation (5) and the communications of the control signals between neighboring nodes.

**Figure 2.**
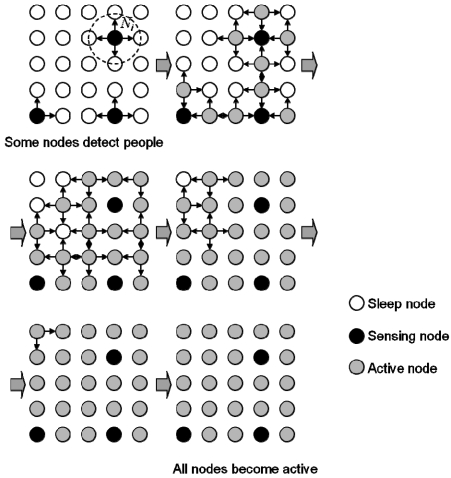
Two-dimensional schematic diagram of signal propagation for activation.

Figure 2 shows the two-dimensional sensor node arrangement and how the signals propagate in the network for activation. To simplify the problem, sensor nodes were arranged at regular intervals to form a mesh network. The dashed circle *N_i_* represents the communication range of sensor node *i*, which covers neighboring nodes. First, the nodes detect people and become active. Then they communicate their signals to neighboring nodes. The nodes that receive the signals become active in turn so that finally all the nodes are active.

**Figure 3.**
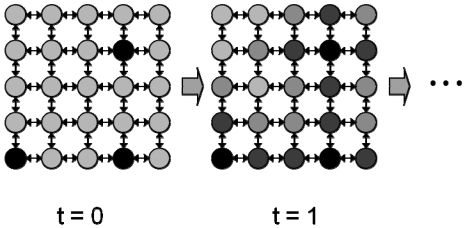
Two-dimensional schematic diagram of control signal computation.

Figure 3 shows how the control signals propagate after activation. They compute their control signals and communicate them to their neighboring nodes in turn. In this way, the control signals are computed as they propagate through the network. After some communication and computation iterations, each node provides the optimal control signal for environment control.

It is very important to reduce the number of iterations in this process. We have to determine a large *ε* value that ensures the stability of Equation (5). Suppose that *m* sensor nodes detect the presence of people. Then Equation (4) is represented as:

(6) $$\text{Hf}-\text{d}=0\;\text{if}\;s_{i}\;=0,$$

where **H** is an (*n-m*) × (*n-m*) matrix, and **f** and **d** are *n-m* element column vectors. The elements of **H** satisfy the following equation:

(7) $${|\text{h}}_{\text{ii}}|\;-\sum_{\begin{array}{l} j=1\\ j\neq i \end{array}}^{n-m}{|\text{h}}_{\text{ij}}|\geq 1-\alpha >0.$$

Then we have:

(8) $${|\text{h}}_{\text{ii}}|\;>\sum_{\begin{array}{l} j=1\\ j\neq i \end{array}}^{n-m}{|\text{h}}_{\text{ij}}|,$$

and **H** is strictly diagonally dominant and regular [14].

In addition, because the diagonal elements of **H** are positive:

(9) $${\text{h}}_{\text{ii}}\;>0$$

**H** is a positive definite matrix.

To guarantee the stability of Equation (5), we have the following equation [12]:

(10) $$0<\varepsilon <2/\lambda_{\max},$$

where *λ*_max_ is the largest eigenvalue of **H**.

With the penalty function method, there are large values in the diagonal elements and large eigenvalues of **H** because the penalty parameter is usually large (e.g. 100) [12]. Therefore, we had to use a small *ε* value, which resulted in the slow computation of *f_i_*. In contrast, we can apply a larger *ε* to Equation (5) than with the penalty function method since we have a smaller *λ*_max_ than that of the penalty function method. This is expected to lead to the fast and precise computation of the control signals.

## 3. Experimental

Crossbow Motes, MICA2DOTs equipped with PIR occupancy sensors (AMN 13112, Matsushita Electric Works, Ltd.) were used as the sensor/actuator network testbed. A MICA2DOT consists of an Atmega128 microcontroller (4 MHz) and a CC1000 radio (315 MHz). The occupancy sensor can be easily attached to the 10-bit A/D converter of the Mote. The sensor reading ranged from 0 to 1023. The software was TinyOS1.1 [15].

**Figure 4.**
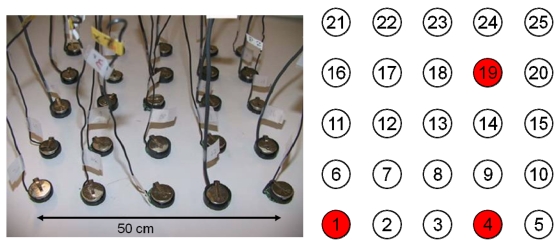
Distributed actuation experiment using Motes.

First, we examined the decentralized algorithm by simulations and by implementing it using Motes. A Mote has digital I/O pins that can control electronic components such as lights. In this experiment, Motes were assumed to detect occupancy by using the PIR sensors and control lights. We assumed that the sensor/actuator nodes were arranged on the ceiling in a shared workplace. The lights are controlled depending on the response of the occupancy sensors. Basically, the occupied areas must be bright and the unoccupied areas may be dark. Any nonuniformity of brightness may be unacceptable to the occupants. Therefore, both energy saving and smoothness of spatial brightness must be taken into consideration. In this experiment, we used 25 Motes, each with an occupancy sensor. Each node's sampling time was 2 s and its neighboring nodes were fixed regardless of the wireless communication range. All nodes were assumed to have a light control unit with a digital output port. Figure 4 shows the experimental setup. Nodes 1, 4 and 19 detected the presence of people and communicated their signals to the neighboring nodes. First we investigated the resulting control signals using the penalty function method. There were 50 communication iterations. Then we evaluated the proposed decentralized algorithm. There were 10 communication iterations.

**Figure 5.**
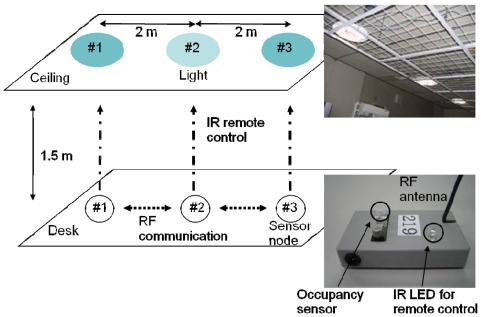
Distributed lighting control experiment using Mote testbed.

**Figure 6.**
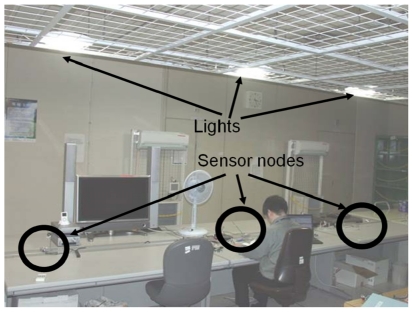
Photograph of the distributed lighting experiment.

Next, we developed a testbed for WSANs based on Motes. The sensor/actuator node consists of a MICA2DOT, a PIC microcontroller (PIC12F629, Microchip Technology Inc.), a PIR occupancy sensor and an IR LED for remote control. The IR LED is driven by the PIC microcontroller and generates remote control signals for lights. The light has an IR detector for reading the control signals. Lighting brightness can vary by eight magnitudes depending on the control signals from the Mote. In this experiment, three lights were deployed on the ceiling and three corresponding nodes were placed on the desk (Figure 5). Each node detected occupancy and communicated the control signal to its neighboring nodes, and then controlled the brightness of a unique light right above it. The Mote's wireless communication range was limited to approximately 2 m. Figure 6 is a photograph of a distributed lighting experiment. First, the three nodes detected three people and set the corresponding lights at maximum brightness. When the two people at nodes 1 and 3 left their desks, the nodes detected the occupancy changes {*s*_1_,*s*_2_,*s*_3_} = {0,1,0} and began to compute the optimal brightness by communicating with each other. In this experiment, the node's communication interval was 5 s and the nodes adjusted the lights every interval. There were 10 communication and control iterations, which means a communication and control duration of 50 s. Experiments were also conducted for the following occupancy changes, {*s*_1_,*s*_2_,*s*_3_} = {1,0,0}, {*s*_1_,*s*_2_,*s*_3_} = {1,0,1}, {*s*_1_,*s*_2_,*s*_3_} = {1,1,0}.

## 4. Results

**Figure 7.**
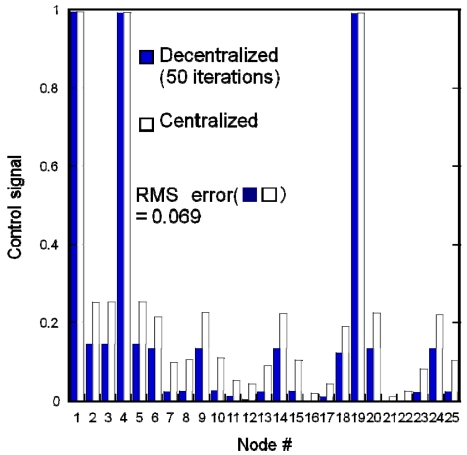
Experimental results of distributed actuation using simulations of centralized and decentralized algorithms. The decentralized algorithm is based on the penalty function method.

Figure 7 compares the control signals simulated by the decentralized and centralized algorithms. Equation (1) was redefined by the penalty function method taking account of Equation (3) [12]. Parameter *α* in Equation (1) was 0.3. The centralized algorithm provides an exact solution for modified Equation (4) by the penalty function method. This result shows that illumination was provided in unpopulated areas close to an area where people were present. In the decentralized algorithm, the initial values of the calculated control signals were determined by sensor responses, that is, 1 for nodes 1, 4 and 19, and 0 for the others. The parameter *ε* in Equation (6) was 0.015. The root mean square (RMS) error between the centralized and decentralized results was 0.069. This result indicates that there were some errors in the estimated control signals when using the decentralized algorithm even with a large iteration number. We must increase the iteration number further to obtain a better estimation.

**Figure 8.**
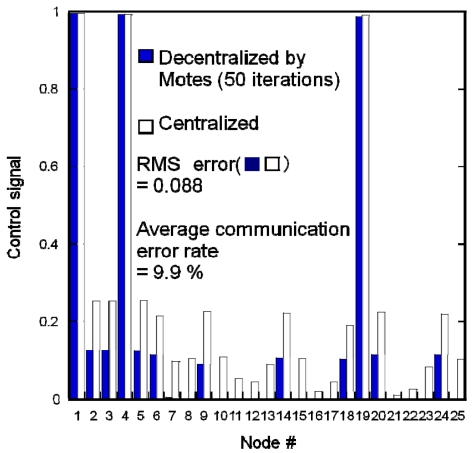
Experimental results of distributed actuation using Motes implemented with decentralized algorithm and simulation of centralized algorithm. The decentralized algorithm is based on the penalty function method.

Figure 8 compares the experimental results for distributed actuation using Motes implemented with a decentralized algorithm and a simulation of the centralized algorithm. The parameters were the same as those used in Figure 7. The Motes were implemented with the same decentralized algorithm as in Figure 7. The RMS error was 0.088. It is clear that, despite some errors, Motes can provide a good estimation. The average communication error rate among the Motes was 9.9%. We believe that communication error led to the Motes' calculation error. When there is no communication error, the Motes can compute perfectly, and provide the same result as that in Figure 7.

**Figure 9.**
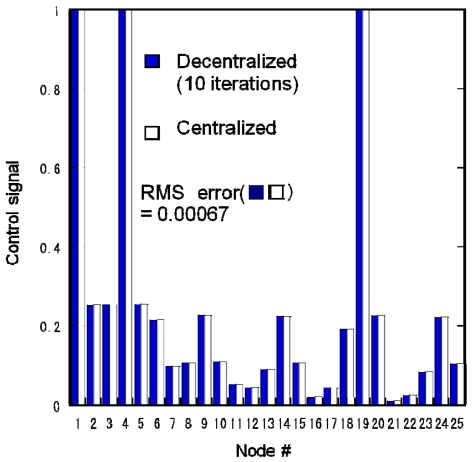
Experimental distributed actuation results obtained using simulations of centralized and proposed decentralized algorithms.

Figure 9 compares the control signals simulated by the proposed decentralized and centralized algorithms. The parameter *ε* in Equation (6) was 0.5 and the iteration number was 10. We were able to use a larger *ε* and a smaller iteration number than in Figure 7. The RMS error was 0.00067, which indicates that this algorithm could achieve better performance than seen in Figure 7. This proposed algorithm enables WSANs to calculate the optimal control signals rapidly and precisely.

**Figure 10.**
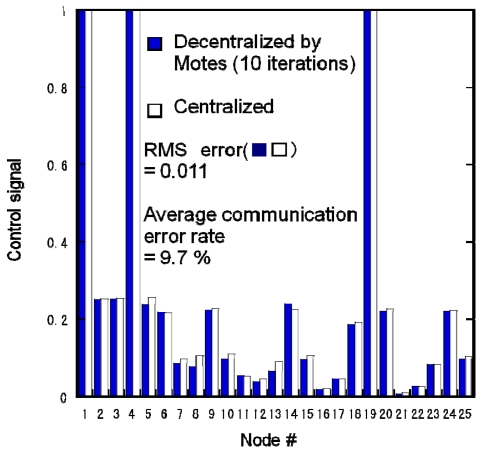
Experimental distributed actuation results obtained using Motes implemented with proposed decentralized algorithm and simulation of centralized algorithm.

Figure 10 compares the calculation results obtained for the Motes and the centralized algorithm by simulation. The parameters were the same as in Figure 9. The Motes were implemented with the proposed decentralized algorithm. The RMS error was 0.011 and the average communication error rate among the Motes was 9.7%. Compared with Figure 8, the RMS error of the proposed algorithm was one-eighth as large as that of the penalty method with one-fifth of its computation time. The computation errors were caused by the communication errors. This result shows that the Motes could provide almost the same result as the simulation in Figure 9. It was found that the algorithm was capable of constructing practical environment control systems using Motes despite the communication errors.

**Figure 11.**
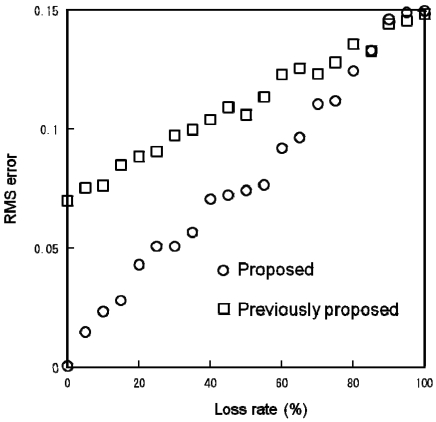
Simulation result of packet loss rate vs. RMS error between centralized and decentralized algorithms (circle: proposed algorithm, square: previously proposed algorithm).

Figure 11 compares the simulation results indicating that how the RMS error depends on the communication error for the proposed and the previously proposed algorithms. The parameters were the same as those in Figures 7 and 9. The Mote's average packet loss rate was approximately 10%. The simulation results indicate that the proposed algorithm is robust against the communication error.

**Figure 12.**
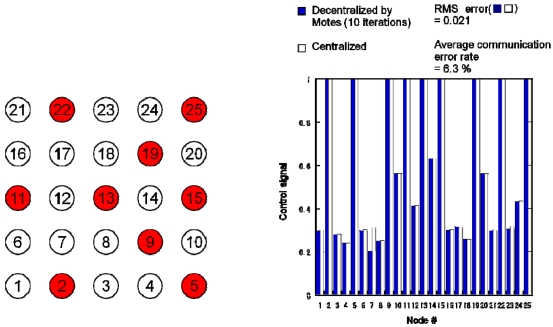
Occupancy pattern and experimental distributed actuation results obtained using Motes implemented with proposed decentralized algorithm and simulation of centralized algorithm.

In order to show that the proposed algorithm can be applied in general cases, we used another occupancy pattern and conducted distributed actuation experiments. Figure 12 shows the occupancy pattern and the distributed actuation results. The Motes could provide a good estimation. This result shows that the algorithm works even if the occupancy pattern changes.

**Figure 13.**
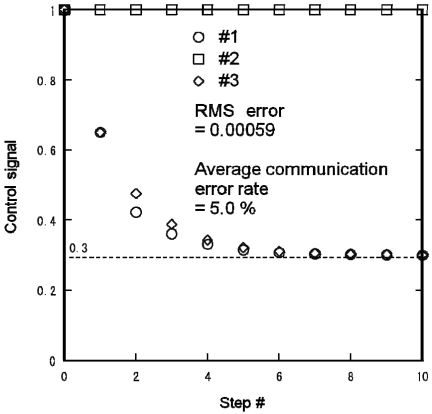
Experimental distributed lighting result obtained using Mote testbed for {s1,s2,s3} = {0,1,0}.

Figure 13 shows the control signal profiles of the three lights in the distributed lighting experiment using a Mote testbed for {*s*_1_,*s*_2_,*s*_3_} = {0,1,0}. The parameters were the same as those in Figure 9. After detecting the occupancy changes, the nodes dimmed the lights gradually and completed computing the brightness at 30% of the maximum brightness for lights 1 and 3. The brightness of light 2 remained unchanged. Using the centralized algorithm of Equation (4), we obtained the control signals of the lights as:

(11) $$\{f_{1},f_{2},f_{3}\}=\{\alpha ,1,\alpha \}=\{0.3,1,0.3\},$$

which are almost the same as those in Figure 11. The RMS error of the calculated control signals was 0.00059 and the average communication error rate among the Motes was 5.0%. This result indicates that the distributed lighting control was performed rapidly and precisely using the Motes implemented with the decentralized algorithm.

**Figure 14.**
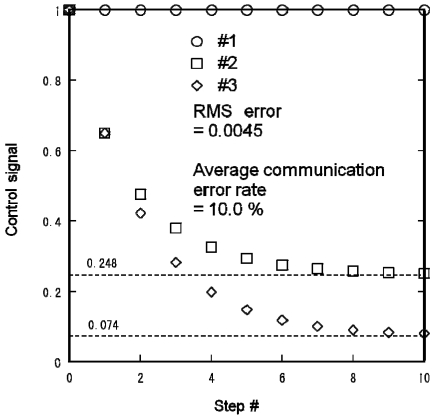
Experimental distributed lighting result obtained using Mote testbed for {s1,s2,s3} = {1,0,0}.

Figure 14 shows the control signal profiles of the three lights for {*s*_1_,*s*_2_,*s*_3_} = {1,0,0}. The control signals calculated from the centralized algorithm are:

(12) $$\{f_{1},f_{2},f_{3}\}=\{1,\alpha /(1+\alpha -\alpha^{2}),\alpha^{2}/(1+\alpha -\alpha^{2})\}=\{1,0.248,0.074\}.$$

The RMS error of the calculated control signals was 0.0045 and the average communication error rate was 10.0%.

**Figure 15.**
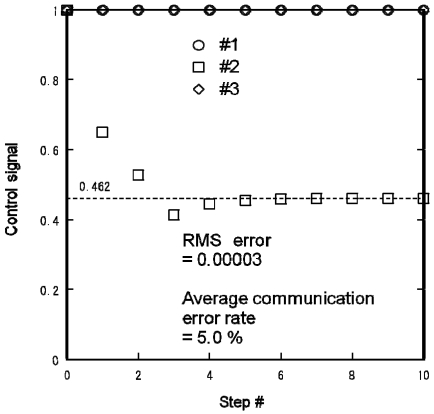
Experimental distributed lighting result obtained using Mote testbed for {s1,s2,s3} = {1,0,1}.

Figure 15 shows the control signal profiles of the three lights for {*s*_1_,*s*_2_,*s*_3_} = {1,0,1}. The control signals calculated from the centralized algorithm are:

(13) $$\{f_{1},f_{2},f_{3}\}=\{1,2\alpha /(1+\alpha ),1\}=\{1,0.462,1\}.$$

The RMS error of the calculated control signals was 0.00003 and the average communication error rate was 5.0%.

**Figure 16.**
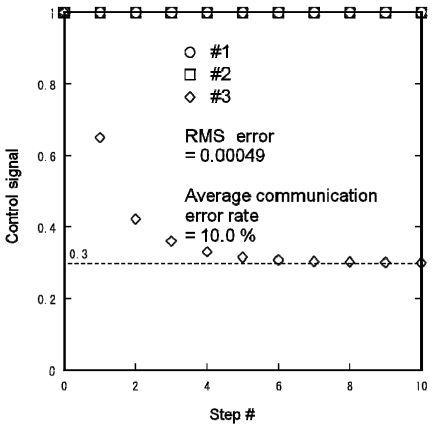
Experimental distributed lighting result obtained using Mote testbed for {s1,s2,s3} = {1,1,0}.

Figure 16 shows the control signal profiles of the three lights for {*s*_1_,*s*_2_,*s*_3_} = {1,1,0}. The control signals calculated from the centralized algorithm are:

(14) $$\{f_{1},f_{2},f_{3}\}=\{1,1,\alpha \}=\{1,1,0.3\}.$$

The RMS error of the calculated control signals was 0.00049 and the average communication error rate was 10.0%.

These results indicate that the various decentralized lighting controls were successful when using the proposed decentralized algorithm of the wireless sensor/actuator nodes. We attained the same accuracy as the centralized algorithm with a small number of communications and computations because of the large value of the step size employed in the iterative process.

## 5. Discussion

The simulation of the centralized collaborative actuation algorithm revealed that optimal control signals can be obtained when the central server can gather data from all the sensor nodes. This algorithm will be useful when it is used in relatively small WSANs. On the other hand, the proposed decentralized collaborative actuation algorithm is more scalable in practical deployment because it does not need a central server. The routing becomes more complicated as the sensor network becomes larger. This algorithm is free from such a complicated routing problem. The trade-off between the accuracy of the calculated signals and the energy consumed by communication is still a significant problem in the decentralized WSANs. We proposed an improved decentralized algorithm for fast control signal computation. Experiments have indicated that the number of iterations is reduced to approximately ten with appropriate step values in the iterative process. We implemented the algorithm on Motes and evaluated its feasibility.

The Mote employing the decentralized algorithm provided the same results as the simulations. The accuracy of computing the control signals depends on the step values, the number of iterations, and the communication quality. However, the increase in iterations leads directly to a long computation time. In addition, communication error is likely to be inevitable in WSANs. Experimental results indicated that using large step values is effective for computing the control signals rapidly and precisely.

The average consensus algorithm [16] is closely related to ours. It minimizes a disagreement function to compute the average of the nodes' initial values. It provides a consensus mechanism to compute the average in a large network. It is also applied to a variety of networks such as dynamic networks. In our work, on the other hand, the aim is not to compute the average but to minimize the sum of the consumed energy and the spatial roughness of brightness. And the topology is fixed in the lighting application. Applying the proposed algorithm to various networks remains to be investigated.

In the proposed distributed actuation model, it is unnecessary to clarify the location of each sensor node. Each sensor node may have access to information about neighboring nodes. It is best to arrange sensor nodes at regular intervals and limit the communication range to their neighbors. One way to recognize neighboring nodes is to use a received signal strength indicator (RSSI). This also remains to be investigated.

The smoothness of controlled signals depends on model parameter *α* When *α* is set at 0, the calculated controlled signal is such that only occupied areas are illuminated and other areas are dark. In contrast, when *α* is set at 1 the calculated control signal is such that all areas are illuminated. The parameter depends on whether the purpose of controlling the environment is to save energy or to maintain user satisfaction.

The distributed lighting experiment showed that the autonomous nodes provided the optimal lighting actuation without a central server. The experiment was on a small scale using only three lights. However, a larger light network would be possible, as shown in Figure 10. These results show that the proposed WSANs are scalable for practical environment control systems.

In this work we assumed that the controlled actuators are mainly lights. Other actuators such as air conditioning systems are applicable, and temperature and humidity can also be controlled. These spatially distributed physical signals are targeted by the proposed WSANs. Our testbed has IR LEDs capable of controlling various devices easily thus constituting a versatile controller. We plan to use it to control other actuators.

In this paper, we developed nodes equipped with sensing and actuation functions. By contrast, it is possible to construct WSANs consisting of three types of nodes: sensors, actuators and controllers [3]. In such networks, sensors are distributed densely, and actuators and controllers are distributed sparsely. We must build a heterogeneous communication model that incorporates these different types of nodes.

As future work, we plan to develop a testbed that adapts to users' actions and preferences. We also plan to employ the proposed algorithm in this testbed and demonstrate distributed sensing and actuation that adapts to the users.

## 6. Conclusions

This paper described improved collaborative sensing and actuation algorithms for providing optimal control of lights in workplaces. In our algorithm, the objective function is defined to balance energy saving against control signal quality. We proposed a decentralized algorithm that can autonomously calculate control signals without a central server. We demonstrated its accuracy and efficiency compared with the previously proposed method using simulations and Motes. It uses appropriate step size values in the iterative process used for calculating control signals. Experimental results showed that it is useful for providing accurate and fast computation because it is difficult to eliminate communication errors. This approach enables practical WSAN deployment because it is easy to implement and maintain. In addition, we described a testbed for distributed lighting control based on the decentralized algorithm. It has IR LEDs for controlling various devices wirelessly and flexibly. It can be used as a versatile controller for other actuators with IR detectors. We showed that it achieved optimal lighting control without a central server. In addition, the decentralized computation was very fast and precise. The experimental results suggest that this approach will allow us to realize a practical environment control system.

## References

[1] Zhao F., Guibas L. (2004). Wireless Sensor Networks: An Information Processing Approach.

[2] Rabbat M., Nowak R. Distributed Optimization in Sensor Networks.

[3] Rabaey J.M. Low-power Sensor Networks.

[4] Robomote.

[5] Coates M., Ing G. Actuator Networks: Distributed Evaluation of Causal Effect.

[6] Taylor K., Ward J., Gerasimov V., James G. Sensor/Actuator Networks Supporting Agents for Distributed Energy Management.

[7] Zhang H., Krogh B., Moura J.M.F., Zhang W. Estimation in Virtual Sensor-Actuator Arrays Using Reduced-Order Physical Models.

[8] Li S. Wireless Sensor Actuator Network for Light Monitoring and Control Application.

[9] Sandhu J.S., Agogino A.M., Agogino A.K. Wireless Sensor Networks for Commercial Lighting Control: Decision Making with Multi-agent Systems.

[10] Singhvi V., Krause A., Guestrin C., Garrett J.H., Matthews H.S. Intelligent Light Control using Sensor Networks.

[11] Lin Y., Megerian S. (2005). Low Cost Distributed Actuation in Large-scale Ad Hoc Sensor-actuator Networks.

[12] Nakamura M., Sakurai A., Furubo S., Ban H. (2008). Collaborative Processing in Mote-based Sensor/actuator Networks for Environment Control Application. Signal Proc..

[13] Bryson A.E., Ho Y.-C. (1975). Applied Optimal Control.

[14] Varga R.S. (1962). Matrix Iterative Analysis.

[15] TinyOS.

[16] Saber R,O., Fax J.A., Murray R.M. (2007). Consensus and Cooperation in Networked Multi-Agent Systems. Proc. IEEE.

